# From Learning to Choosing: How Decision-Making Evolves with Experience in Rats

**DOI:** 10.1523/ENEURO.0270-24.2024

**Published:** 2024-07-16

**Authors:** Kendra M. Loedige, Mohammed U. Al-youzbaki

**Affiliations:** ^1^Departments of Neuroscience, Western University, London, Ontario N6A 3K7, Canada; ^2^Anatomy and Cell Biology, Western University, London, Ontario N6A 3K7, Canada

Decision-making is a fundamental process that guides actions by selecting between various options based on their known or presumed outcomes, often using sensory inputs (Carandini and Churchland, 2013). The neural mechanisms by which the brain integrates complex information to make decisions are typically studied by measuring neural recordings, response times, and choice accuracy using well-trained animals (Carandini and Churchland, 2013). These experiments often employ two-alternative forced-choice (2AFC) designs, where animals are trained to choose between two stimuli presented simultaneously, assuming that the animal learns the value of each stimulus and decides based on an internal comparative evaluation.

However, the traditional 2AFC design may be limited, as it does not consider that stimuli are not encountered simultaneously in nature and that decision-making strategies evolve during learning (White et al., 2024). Kacelnik et al. (2011) highlight these concerns, arguing that animals’ choices on a 2AFC task could be predicted by latencies observed when animals are provided with a single offer. Furthermore, they suggest that the act of deliberation, observed when animals slow their response times to make a choice, could be an artifact produced as animals learn the 2AFC.

The recent study by White et al. (2024) in *eNeuro* aimed to address these limitations by investigating decision-making dynamics during the initial learning phase. By isolating learning values of individual stimuli from the decision-making process, the researchers sought to understand how male rats can make choices for the first time on a 2AFC and how those choices change with experience.

**Figure 1.**
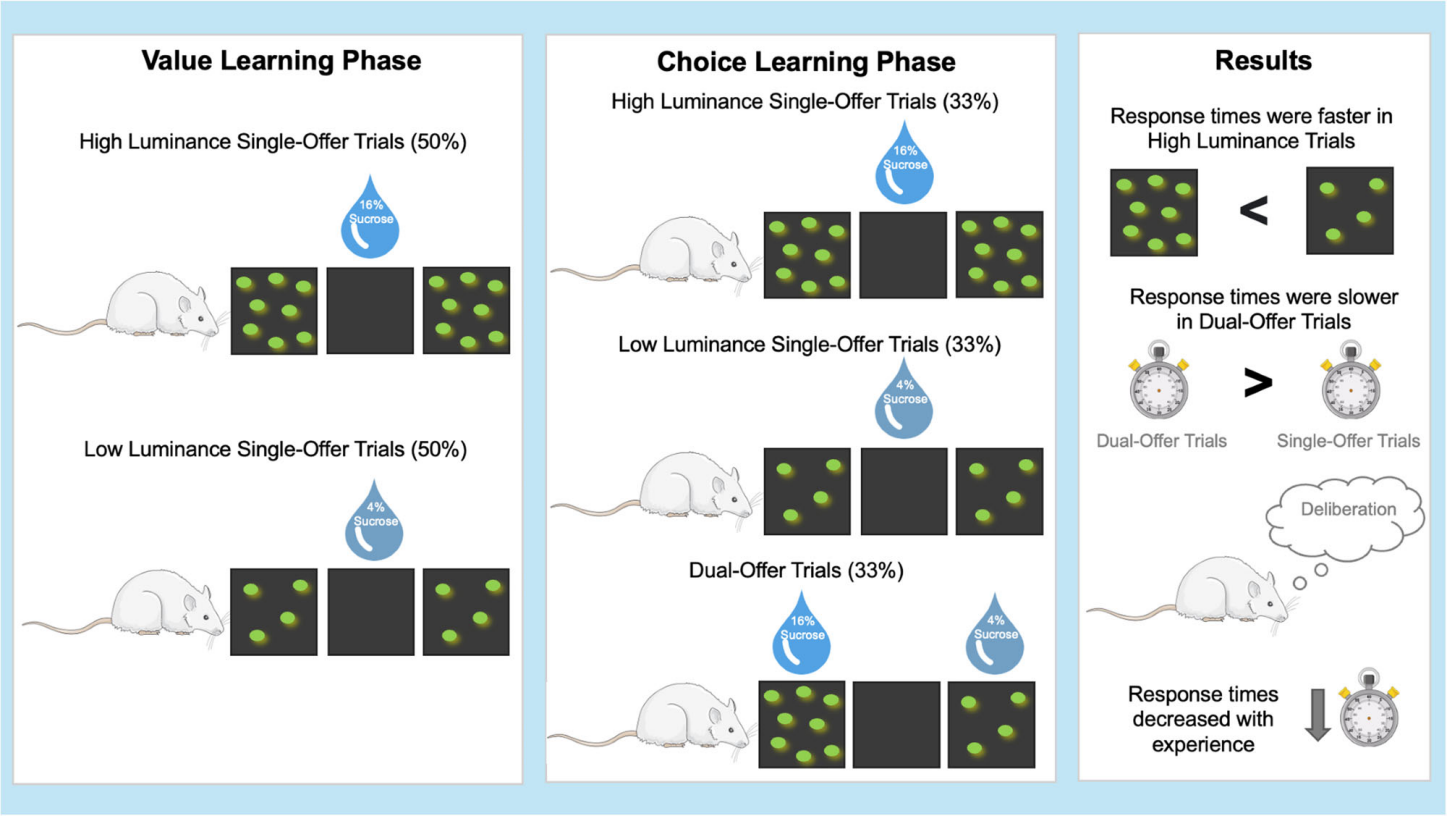
Visual abstract of the experimental design and findings by White et al. (2024), separating reward value learning from the decision-making process. During the value learning phase, rats were introduced to single-offer rewards. They initiated trials by nose-poking in the central nose port, which presented a visual stimulus through LEDs on either the left or right side. The visual stimuli were equally split between high luminance (high reward, 16% sucrose) and low luminance (low reward, 4% sucrose). In the subsequent choice learning phase, trials were divided equally among high-reward, low-reward, or dual-offer trials, where male rats had to choose between stimuli of high and low luminance. The results indicated that rats responded faster to high-luminance stimuli than low-luminance stimuli in single-offer trials. Response times were slower in dual-offer trials compared with single-offer trials, suggesting a deliberation process affecting decision-making. Response times decreased with experience, demonstrating the effect of initial learning on subsequent decision-making [adapted from White et al. (2024)].

The behavioral experiment comprised two main stages: value learning and choice learning (Fig. 1). In the value learning stage, rats were introduced to a single stimulus of either high or low luminance, where nose pokes at the port below the stimulus led to the delivery of a high (16% sucrose) or low (4% sucrose) reward, respectively. The choice learning stage consisted of single-offer trials for two-thirds of the total trials, where rats were presented with either the high or low-luminance cue. The remaining one-third of trials were dual-offer trials, where animals were simultaneously offered both high- and low-luminance stimuli, randomized by side. Rats’ response latencies and choice percentages were measured. During the initial stages of choice learning, rats consistently preferred the high-luminance stimuli. Median response latencies for dual-offer trials were greater compared with single offers and were greater for single-offer trials with low-value stimuli compared with those with high-value stimuli. These latency differences were most pronounced in the first session but persisted over the remaining sessions. These differences indicate that even after dissociating the value learning stage from the decision-making process, rats showed evidence of deliberation, which remained present with experience.

To examine changes in the response time distribution that occur with experience, the authors utilized an ExGauss fitting. In this model, response times are fitted with Gaussian and exponential components, considering the peak and tail of the response time distribution, respectively. This enables the quantification of sensorimotor processing (Gaussian component) and variability (exponential component) within the data. The fitting showed that when choosing the high-value reward, there was increased variability in dual-offer trials compared with single-offer trials. Although the overall variability in high-reward trials decreased with experience, response time variability remained higher in dual-offer trials.

Behavioral data was further analyzed using drift diffusion modeling (DDM), a commonly used computational model within the decision-making field. The DDM assumes that when faced with a binary decision, such as between high and low rewards, noisy evidence accumulates with time favoring one of the two options. When enough evidence is reached for one option, it crosses a threshold, allowing for a decision to be made (Myers et al., 2022). DDM revealed that initial task learning lowered the decision threshold, meaning rats required less evidence to choose between high and low rewards. Interestingly, the decision threshold of the rats was highly correlated with the variability observed in the ExGauss fitting. As discussed by the study authors, this relationship suggests that lowering the decision threshold with experience could form a more reliable decision-making system, resulting in decreased variability in response times.

White et al. (2024) provide novel insights into how rats adapt their decision-making processes through learning using a two-stage experimental design, demonstrating that this process does not rely solely on fixed strategies. The differences in latencies observed during the value learning stage between high- and low-luminance stimuli indicate the differentiation between and encoding of reward values during initial learning. When transitioned to the choice learning stage, rats initially exhibited longer response latencies in dual-offer trials compared with single-offer trials, suggesting the rats deliberate when choosing between options of known value. These latencies persisted at an attenuated level over the remainder of the choice learning sessions, suggesting that experience shapes decision-making performance.

Importantly, this finding contrasts with Kacelnik et al. (2011), who suggested that animals do not deliberate when making simultaneous choices but rely on the same processes as when options are presented sequentially. The findings by White et al. (2024) suggest that rats do engage in comparative evaluation when faced with simultaneous choices and that this process evolves during the learning phase.

The observation of deliberation processes in rats marks a significant stride forward in understanding how decision-making evolves with experience. To better elucidate the decision-making processes in different contexts, future studies can alter stimulus complexities and reward levels, introducing rats to varying decision difficulties. Additionally, it is worthwhile to investigate if female rats demonstrate similar results, as prior studies have demonstrated sex differences in the learning period of decision-making (Chen et al., 2021). Evaluating decision-making in this context across sexes and using other animal models could enrich our understanding of decision-making processes across different biological systems. Ultimately, this study underscores the importance of experimental designs reflecting the natural sequential encounter of stimuli and considering how learning may influence decision-making strategies. By employing such approaches, the neural and cognitive mechanisms underlying decision-making can be better understood.
